# Editorial: Liquid biopsies in hematological malignancies

**DOI:** 10.3389/fimmu.2024.1440394

**Published:** 2024-06-12

**Authors:** Susana García-Silva, Dario Marchetti, Miguel Gallardo

**Affiliations:** ^1^ Microenvironment and Metastasis Laboratory, Molecular Oncology Programme, Spanish National Cancer Research Center (CNIO), Madrid, Spain; ^2^ Departments of Internal Medicine and Pathology, The University of New Mexico, Health Sciences Center, Albuquerque, NM, United States; ^3^ Department of Hematology, Hospital Universitario 12 de Octubre, Instituto de Investigación Sanitaria Hospital 12 de Octubre (imas12), Madrid, Spain

**Keywords:** liquid biopsies, hematological malignancies, minimal residual disease (MRD), immunotherapy, next-generation sequence (NGS)

A liquid biopsy is a non-invasive diagnostic procedure which involve sampling and analyzing body fluids, primarily blood, to detect and monitor various diseases. This approach allows for the versatile evaluation of genetic mutations, cell-free DNA quantification and properties (cfDNA), circulating tumor DNA (ctDNA), circulating tumor cells (CTCs) and other biomarkers (1–3). Liquid biopsies facilitate real-time monitoring of disease progression and treatment response in a non invasive way, making it a valuable tool in personalized medicine, minimal residual disease (MRD) evaluation and early cancer detection (4–6).

Hematologists and cancer biology researchers have been pioneers in the research and development of blood-based biomarkers, mostly due to the fact that the primary biological material of analysis in hematology is the blood itself (7, 8). In addition, the presence of disease in blood for many hematologic malignancies makes it a perfect and direct testing ground for the development of liquid biopsy techniques and MRD analyses (9, 10).

Therapy monitoring, and in particular, next‐generation immunotherapies like checkpoint inhibitors, bispecific immunomodulatory antibodies and chimeric antigen receptor T‐cell therapy (CAR‐T), pose challenges regarding the identification of meaningful biomarkers for patient selection and follow-up of treatment regimes. In this context, liquid biopsy-based approaches can rapidly provide useful information for clinical decisions (11–13).

The publications of this Research Topic have generated two significant insights: first, the great interest in immunotherapy treatments, and the importance of predicting patient response together with disease status at each precise moment of the treatment. Second, this Research Topic has addressed some of the most common questions and pitfalls in the development and application of techniques for liquid biopsy analysis:

1. The lack of complex algorithms that combine the new MRD analysis techniques (e.g., next-generation sequencing or NGS) with the classical ones (e.g., PET/CT, multiparametric flow cytometry). These challenges are described by Jimenez-Ubieto et al. or Zhao et al.
2. The lack of standardization of specific techniques and their adaptation for liquid biopsy. These problems have been described in the work of Peng et al. and Jiang et al. The need for consensus criteria in genomic analyses by next-generation sequencing is particularly glaring.

In: “Personalized monitoring of circulating tumor DNA with a specific signature of trackable mutations after chimeric antigen receptor T-cell therapy in follicular lymphoma patients”, Jimenez-Ubieto et al. analyzed follicular lymphoma patients with anti-CD19 CAR T-cell therapy and identified patient-specific genetic alterations, following mutation dynamics after treatment using the novel LiqBio-MRD technique, a type of ultra-deep NGS. They combined the results with traditional MRD analysis, PET/CT. The work demonstrated that a non-invasive liquid biopsy MRD analysis may correlate with response and could be used to monitor response in CAR T-cell therapeutic approaches.

Conversely, in: “From rough to precise: PD-L1 evaluation for predicting the efficacy of PD-1/PD-L1 blockades”, Zhao et al. discussed about the relevance of determining PD-L1 profiles in both tumor and non-tumor, immune and non-immune compartments. They highlighted the importance of combining different novel PD-L1 detection techniques, such as PET and single-photon emission computed tomography (SPECT), for analyses of PD-L1 expression dynamics, as well as the use of liquid biopsy approaches for evaluating PD-L1 in circulation. The review offered an interesting comparison between the different proposed techniques and stated the potential contribution of artificial intelligence for the correct evaluation and quantification of PD-L1 levels through the integration of different input values.

In: “A Support Vector Machine Based on Liquid Immune Profiling Predicts Major Pathological Response to Chemotherapy Plus Anti-PD-1/PD-L1 as a Neoadjuvant Treatment for Patients With Resectable Non-Small Cell Lung Cancer”, Peng et al. analyzed stage Ib-IIIa NSCLC patients undergoing neoadjuvant chemotherapy plus anti-PD-1/PD-L1 (CAPD) before surgical resection. They built a model combining support vector machine (SVM), a machine learning system, together with liquid immune profiling (LIP-SVM), a multiparametric cytometry system. The authors developed a predictive model for the major pathological response of patients with NSCLC undergoing CAPD treatment that can potentially guide clinical therapy.

In: “Exploring biomarkers for prognosis and neoadjuvant chemosensitivity in rectal cancer: Multi-omics and ctDNA sequencing collaboration”, Jiang et al. analyzed the efficacy of neoadjuvant chemotherapy by DNA sequencing in rectal cancer patients. The authors presented the combination of a large battery of genomic techniques: single nucleotide variants (SNV), copy number variation (CNV), tumor mutation burden (TMB), copy number instability (CNI) and mutant-allele tumor heterogeneity (MATH). This work demonstrates the effectiveness of integrative analyses of multi-omics data for the identification and use of robust biomarkers.

In summary, this Research Topic highlights the need for greater transparency in the protocols, biological material, techniques and algorithms used by researchers. It also emphasizes the need for increased collaboration between consortia and international groups, setting aside the economic conflicts of interest that always arise in the development of new technologies, ultimately benefiting the patient.

## Author contributions

SG-S: Writing – original draft, Writing – review & editing. DM: Writing – original draft, Writing – review & editing. MG: Conceptualization, Writing – original draft, Writing – review & editing.
